# Therapeutic Fc fusion protein misfolding: A three-phasic cultivation experimental design

**DOI:** 10.1371/journal.pone.0210712

**Published:** 2019-01-16

**Authors:** Atefeh Ghorbani Aghdam, Saeed Moradhaseli, Farnoush Jafari, Paria Motahari, Sepideh Samavat, Rasoul Mahboudi, Shayan Maleknia

**Affiliations:** Biopharmaceutical Research Center, Aryogen Pharmed Inc., Alborz University of Medical Science, Karaj, Iran; The University of Texas at El Paso, UNITED STATES

## Abstract

Cell culture process optimization is a critical solution to most of the challenges faced by the pharmaceutical manufacturing. One of the major problems encountered in large-scale production of therapeutic proteins is misfolded protein production. The accumulation of misfolded therapeutic proteins is an immunogenic signal and a risk factor for immunogenicity of the final product. The aim of this study was the statistical optimization of three-phasic temperature shift and timing for enhanced production of correctly folded Fc-fusion protein. The effect of culture temperatures were investigated using the biphasic culture system. Box–Behnken design was then used to compute temperature and time of shifting optimum. Response surface methodology revealed that maximum production with low level of misfolded protein was achieved at two-step temperature shift from 37°C to 30°C during the late logarithmic phase and 30°C to 28°C in the mid-stationary phase. The optimized condition gave the best results of 1860 mg L^−1^ protein titer with 24.5% misfolding level. The validation experiments were carried out under optimal conditions with three replicates and the protein misfolding level was decreased by two times while productivity increased by ~ 1.3-fold. Large-scale production in 250 L bioreactor under the optimum conditions was also verified the effectiveness and the accuracy of the model. The results showed that by utilizing two-step temperature shift, productivity and the quality of target protein have been improved simultaneously. This model could be successfully applied to other products.

## Introduction

Nowadays, recombinant Fc-fusion protein production presents an attractive technique for development of new protein-based therapies against several chronic diseases including rheumatoid arthritis, platelet disorders and psoriasis [1, 2]. The Fc domain of such therapeutics, responsible for the hybrids prolonged half-life, is commonly coupled to a therapeutic protein and extends a protein's *in vivo* half-life via the Fc receptor recycling mechanism [3, 4].

Chinese hamster ovary (CHO) cells are the preferred host expression system for Fc-fusion protein production whilst six out of nine Fc-fusion molecules on the market are expressed in CHO cells [1]. This is mainly due to the ability of CHO cells to perform complex post-translational modifications (PTMs). These modifications are crucial for full function of therapeutic products. The abolishment of PTMs may disrupt folding pathway [5].

Protein misfolding can be induced by a number of culture process parameters such as temperature, pH or protein biochemical modifications, including deamidation, oxidation and glycation [6]. It is further reported that misfolded proteins are produced during cell culture process and it can consist of more than 50% impurities at the end of the cell culture process, which leads to considerable yield decrements in commercial-scale manufacturing [7].

Furthermore, due to the biological activity and safety concerns, regulatory agencies mandate that recombinant therapeutic proteins should be free of impurities that can trigger immunogenicity. So, one of the major complicating factors in large-scale product development is elimination of product and process related impurities. Previous studies indicated that misfolded proteins might maximize the risk of immunogenic signal, explaining why reducing misfolded isomers of therapeutic proteins, as one of the protein-related impurities, is one of the main goals to build quality in final product [8].

Upstream process contains some operating parameters that if being optimized, they will contribute to high product yield while meeting the quality specifications. Amongst all the parameters, temperature has a significant impact on accurate protein folding. In addition to potential benefits on protein folding, applying sub-physiological temperatures during cell culture process especially in production phase has also been reported to increase the cell-specific productivity [9]. Marchant and his colleagues reported the cell-specific productivity (qMab) of IgG4 monoclonal antibody (cB72.3) as increased by 50% after temperature shift to 32°C from 37°C during the stationary phase of growth [10]. It is quite in contrast with the improved production of anti-4-1BB antibody, which is highly suppressed at lower temperature (30, 33 degrees C) [11].

Furthermore, the time point of culture temperature shifting can also affect cell proliferation rate and productivity. These two parameters should be set in a way to effectively decrease the protein misfolding while minimizing the effect of temperature downshift on cell growth and viability. The culture temperature can be switched gradually or in discrete intervals while the culture shifts from growth phase to production phase [12]. The gradually decrease in temperature may result in a much better performance of cells regarding growth rate and viability while improving the protein folding at lower culture temperatures. To investigate the role of culture temperature on product yield and production of misfolded protein in a specific CHO cell line, we first examined the effect of low culture temperatures in the range of 28–32°C. Lowering the temperature had been shown to be beneficial for protein folding, while being detrimental for production yield. To provide insight into the conditions with higher yield and quality, two-step temperature downshift was then employed.

The conventional method of process optimization, one factor-at-a-time, is time-consuming, expensive, and often leads to misinterpretation of results when interactions between different components are present. Statistical experimental designs minimize the error in determining the effect of parameters and it also allows variation of all parameters. Response surface methodology (RSM) is commonly used for statistical optimization of various industrial process parameters. It is an effective mathematical strategy that can explore the interactions between several factors [13]. The Box-Behnken design (BBD) of the RSM with fewer experiments is an efficient tool to assess the effect of operation variables in a shorter cycle time [14, 15].

This study was performed to describe a two-step thermal shift to optimize conditions for higher expression and more accurate rate of protein folding by means of BBD.

## Materials and methods

### Cell line and culture medium

The suspension CHO cell line producing a 150 KDa fully humanized tumor necrosis factor receptor Fc-fusion protein from Research Working Cell Bank (RWCB) of Aryogen Pharmed (Alborz, Iran) was used. The original source of CHO DG44 ordered from Thermo Fisher Scientific, /Gibco, USA (Catalog no: A10971-01). The culture medium was ActiCHO P (GE Healthcare) supplemented with 6 mM L-glutamine (Lonza, Verviers, Belgium) and related feeds (feed A and B, GE Healthcare) at the moment of preparation as recommended by the supplier. Cells were propagated using 500-ml baffled shake flask containing 100-ml culture media incubated on an orbital shaker with 75 RPM (30 mm amplitude of rotation, Witeg, Germanny) at 37°C with 5% CO2.

### Fed batch culture

The experiments were performed as fed-batch cultivation in 5-L STR bioreactor (Biozeen, India) equipped with 3-segment blade impellers. The cultures were seeded at 6± 0.5 × 10^5^ cells mL^-1^ with an initial working volume of 3.5 L. Cells were cultured at 37°C and subsequently shifted down to new set points according to the experimental design. Agitation speeds was set at 140 RPM (P/V: 0.034 W/L) and pure oxygen-sparging was used for aeration in order to maintain dissolved oxygen (DO) level at 60% of air saturation. pH was maintained at 7± 0.2 by using CO_2_ sparging and 1N sodium hydroxide. The cultures were fed daily (starting on day 2) with the corresponding ActiCHO Feed A and Feed B in a way to keep glucose concentration between 3–5 g/L. Process parameters such as cell density, viability, osmolality, glucose uptake and lactate production were monitored daily. All batches were terminated on day 15 of culture.

### Analytical methods

Cell density and Viability were measured using the trypan blue exclusion-based method (Sigma, Oakville, Canada,). Osmolality was measured using Osmomat 3000 cryoscopic osmometer (Genotec, Germany). Glucose and lactate concentration were analyzed using a Biosen lactate and glucose Analyzer (EKF Diagnostics, Germany). The protein expression during the cell culture process was determined through in-house developed enzyme linked immunosorbent assay (ELISA). The final protein concentration at the end of batch was determined by MabPac Protein A affinity column (Thermo scientific, CA, USA).

Cell specific productivity, (Qp), was measured in pg/cell/day during growth phase using the following equation:

$$Qp:\frac{Titer}{IVCC}$$

Where IVCC (cell-day/ml) is the integral of cell concentration and calculated as:

$$IVCC_{t:}\frac{VCD(t)+VCD(t-1)}{2}\times \frac{\Delta t}{24}+IVCC\;(t-1)$$

Where VCD is the viable cell density (x10^6^ cells.ml^-1^) at a given time and Δt means a change in time since the previous time point [16].

### Fusion protein purification and quantification

Fc-fusion protein was purified from clarified culture supernatant by protein A column operated on a AKTA PURE system (GE Healthcare). The protein A capture step was performed with an XK16/20 column filled with MabSelect SuRe LX resin (GE Healthcare) with phosphate-buffered saline (PBS) equilibration followed by loading and washing with PBS. The protein was then eluted with 100 mM sodium citrate at pH 3. The protein quantification was performed using UV/Vis spectrophotometer (Lambda 25, Perkin Elmer) at 280 nm wavelength.

### Identification of misfolded variants

The hydrophobic interaction chromatography-high performance liquid chromatography (HIC-HPLC) is a critical technique for molecular variant characterization of therapeutic proteins [17]. The mixture containing the target protein in correct and misfolded forms were contacted with the HIC adsorbents under the salt gradient conditions to selectively release the protein. 10μl of sample was injected for separation. HIC-HPLC utilized TSKgel Butyl-NPR (4.6 mm ID × 10 cml, TOSOH Bioscience) reverse phase column and a (NH_4_)_2_ SO4-Na_2_HPO_4_/Na_2_HPO_4_ (pH: 7.0± 0.1) gradient system to determine the relative molecular purity of product. Sample was eluted with a linear gradient starting at 100% mobile phase A 1.8 M (NH_4_)_2_ SO4- 0.1 M Na_2_HPO_4_ and a flow rate of 1 mL/min, transitioning to 100% mobile phase B over a course of 50 min (mobile phase B: 0.1 M Na_2_HPO_4_ at pH 7.0± 0.1) and then to 100% mobile phase A over 1 min before re-equilibration of 100% mobile phase A. The total run was 70 min. Absorbance at 214 nm was recorded at the column exit. The calculation of relative percentage area under each peak refers to the percentage of each species in the sample.

### Preliminary thermal shift study

Biphasic temperature shift experiments were applied to evaluate the impact of single temperature shift on the quality and quantity of the product. Fed-batch experiments were carried out in 5-L stirred tank bioreactor which was operated in standard condition at 37°C with different temperature downshifts (32°C, 31°C, 30°C, 29°C and 28°C) to investigate the level of misfolded proteins as well as the final protein titer. The temperature was shifted down in the middle of logarithmic phase (120 h). Furthermore, biphasic cultures involving a shift in temperature to 30°C or 28°C during the log phase (days 3, 5 and 7) were conducted to evaluate the effect of time point of culture temperature shifting on protein expression and misfolding levels.

### Experimental design and statistical analysis

Three-phasic cultivation method was followed for the optimized expression of the protein according to the statistical approach. To analyze the effects of three independent variables (first temperature shift (factor A), first and second temperature shift time (factor B and factor C (respectively) on fusion protein expression and misfolding levels (responses) in CHO cells, response surface methodology was carried out based on the Box–Behnken factorial design scheme. The Software package Design Expert version 7.1.2 (Statsoft, USA) was used to find out the optimal temperature shift and timing. In all experiments the second temperature downshift was adjusted on 28°C. Each variable was coded as *−*1 (low), 0 (central point) and *+*1 (high) (Table 1). The center point was repeated five times to provide a good estimate of the experimental error [18]. The Box–Behnken design with a total of 17 runs was used to obtain a quadratic model. The experimental results were analyzed and modeled using a second-degree polynomial model as Equation:

Y=β0+β1X1+β2X2+β3X3+β4X1X2+β5X1X3+β6X2X3+β7X1X1+β8X2X2+β9X3X3

where *Y* is the predicted responses (productivity (mg L^−1)^) and misfolding level (%)); X1, X2 and X3 are the coded independent factors; *β*0 is the intercept parameter; *β*_1_, *β*2 and *β*3 correspond to the linear coefficients; *β4*, *β5* and *β6* are the coefficients of interaction effects; and *β7*, *β8*, and *β9* are the coefficients for quadratic terms.

**Table 1:** The values of independent variables and corresponding levels chosen for trials in Box-Behnken experimental design.

Factors	-1	0	+1
First temperature downshift (°C)	30	31	32
Time of 1^st^ temperature shift (h)	72	120	168
Time of 2^nd^ temperature shift (h)	192	240	288

The adequacy of the models were evaluated by applying the *R*^*2*^ statistic (coefficient of determination), response plots and analysis of variance (ANOVA). In addition, model terms were selected or rejected according to the calculated probability value with 95% confidence levels. Surface plot (three-dimensional) and corresponding contour plots (two-dimensional) were provided for productivity and misfolding level based on the effects of three independent variables.

### Validation and verification of the model in pilot scale experiments

Validation of the predicted model was carried out by performing three consecutive batches in 5L scale bioreactors and analyzed using Tukey's multiple comparison test. Then, the resulting optimized condition was used to verify the model in 30 and 250 L bioreactors (Biozeen, India) for three independent runs, according to the constant impeller P/V = 0.034 criterion. The other process parameters remained the same as the 5 L bioreactor.

## Results

### Preliminary temperature shift assay (biphasic cultivation): Growth kinetics

**Fig 1:**
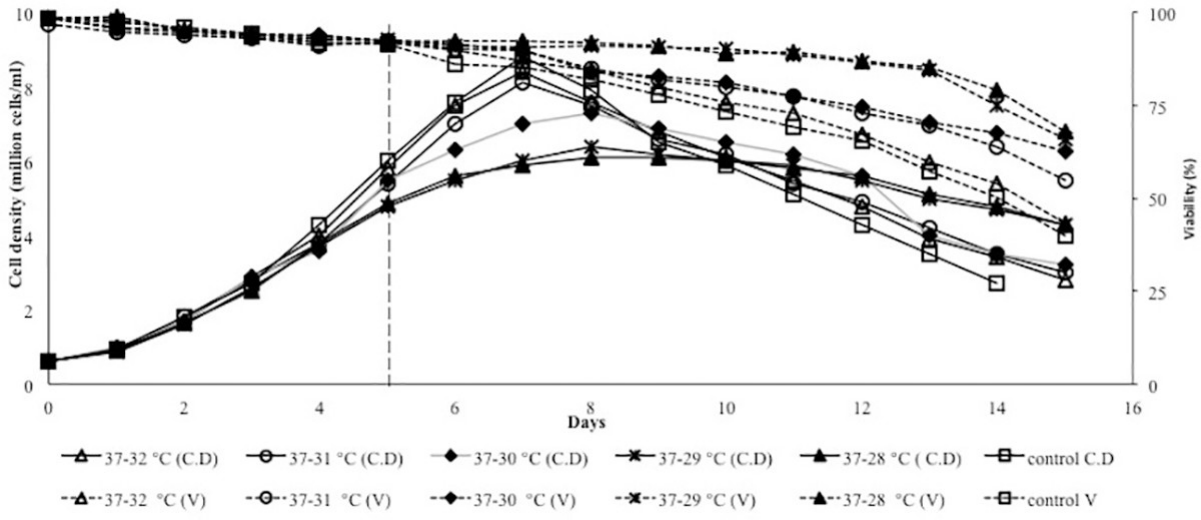
Kinetics of cell growth. Cell density of Fc-fusion producing CHO cell line (continues lines). Viabilities of Fc-fusion producing CHO cell line (dashed lines). Control condition was carried out at 37°C. (□, Control culture; Δ, shift to 32°C; ○, shift to 31°C; ♦, shift to 30°C; *, shift to 29°C; ▲, shift to 28°C. Vertical dashed line indicates the time of temperature shift.

Preliminary biphasic culture experiments were conducted to study the differences in cell growth in response to temperature downshifts in the range of 32°C-28°C. Thus, cell culture kinetics were investigated in fed batch mode cultures in 5-L scale bioreactor. Maximum viable cell concentration (Max VCC) was not greatly affected for temperature shift to 32°C, but shifting to 30°C, 29°C or 28°C resulted in decreased Max VCC compared to the control condition, reaching a peak of 7.3×10^6^ cells/ml, 6.4×10^6^ cells/ml and 6.1×10^6^ cells/ml, respectively (Fig 1). The maximum cell density achieved in the control culture was 8.8×10^6^ cells/ml. Thus, a reduction by 30% is observed in peak cell density of the culture carried out at 28°C.

Cell viability profiles were also different between the cultures. Almost all cultures reached a viability peak of ~ 90% at day 7, however this would contradict the lower viability observed towards the end of the culture. As shown in Fig 1 shifting to a lower temperature promotes viability maintenance, although there is no significant differences in their culture durations. The differences observed after day 9, where the cultures with temperature shift to values higher than 30°C (37–32°C, and 37–31°C) and the control condition appeared to lose viability more rapidly than those with temperature shifted cultures to 29°C and 28°C (at day 10 viability was 90% in conditions with temperature downshift to 29°C and 28°C however, viability decreased to 81%, 80%, 75% and 73% in cultures shifted to 30°C, 31°C, 32°C and the condition without temperature shift, respectively).

Table 2 shows the cell specific growth rate (μ) and integral of viable cell concentration (IVCC). The μ values decreased slightly during a temperature shift to a value below 30°C and almost constant regardless of the temperature shift to 32°C and 31°C, whereas the IVCC was affected by the culture temperature and the highest value was achieved during a temperature shift to 32°C. Reduced specific growth rate resulted in lower IVCC in conditions with temperature shift to the values below 30°C (Table 2).

**Table 2:** Process relevant data from preliminary fed-batch cultures. (μ is the average value of specific growth rate for two days after shifting the temperature).

Cell-culture Temperature	37°C	37 °C-32 °C	37°C-31°C	37°C-30°C	37°C-29°C	37°C-28°C
Max. VCC (10^6^ cells/ml)	8.8±0.02	8.4±0.03	8.1± 0.05	7.3± 0.03	6.4±0.02	6.1±0.03
IVCC	68.78±1.52	72.92±0.88	71.66±0.98	71.56±1.00	68.26±0.77	68.19±0.65
Batch duration (day)	14	15	15	15	15	15
μ (specific growth rate) (h^-1^)	0.010± 0.0002	0.010± 0.0002	0.010± 0.0002	0.009± 0.0007	0.007± 0.0002	0.006± 0.0005
Protein final titer (mg/L)	1406±45.25	1500±41.71	1530±24.74	1600±73.53	1350±20.5	1100±29.69
Qp (pg/(cell×day))	20.44±0.14	20.57±0.22	21.35±0.03	22.35±0.73	18.86±0.4	16.13±0.41
Misfolding level (%)	48±0.63	46±0.91	42±0.63	33±0.63	31±0.98	23±0.14

**Fig 2:**
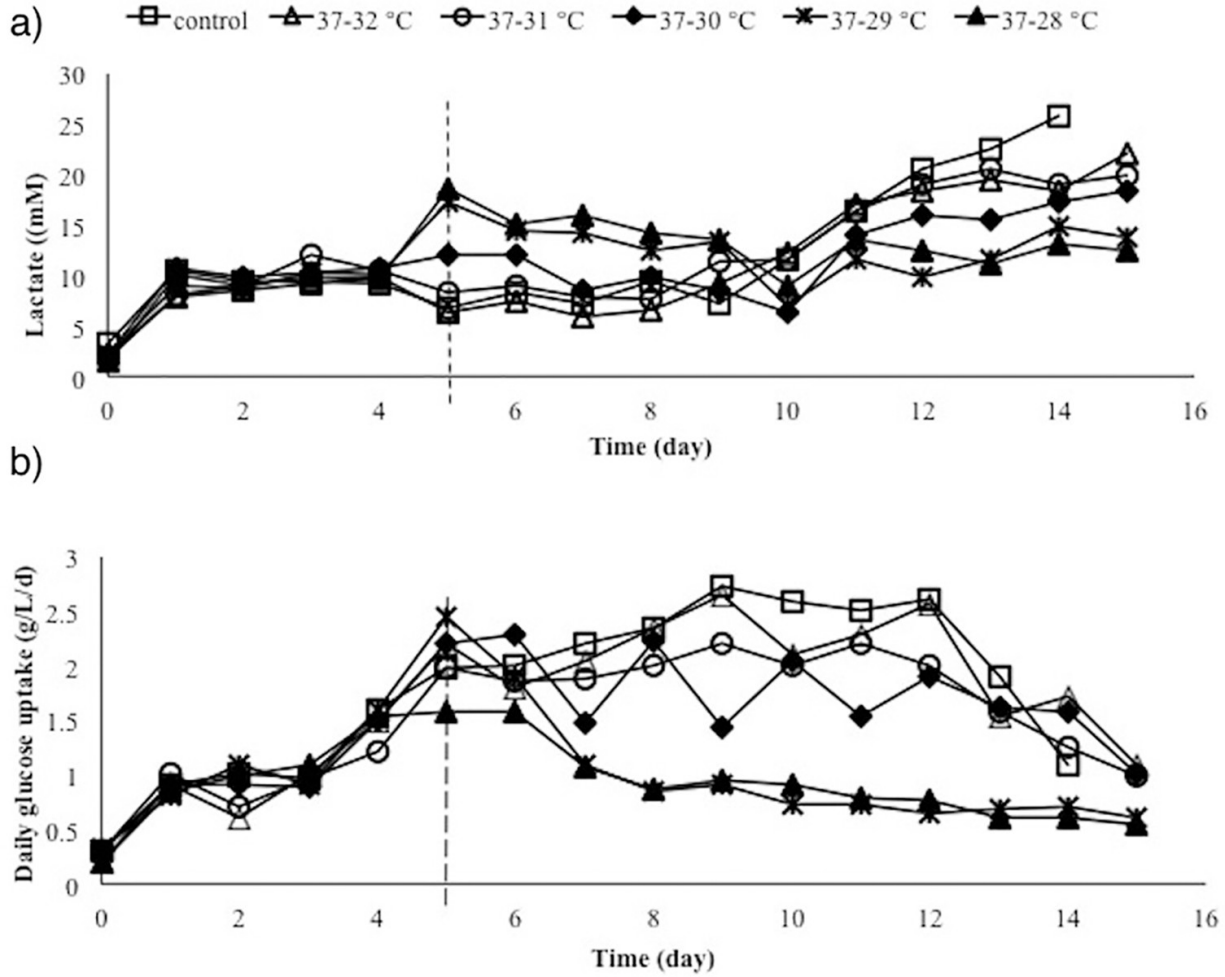
a) Lactate levels (mM) and b) daily glucose uptake rate (g/L/day) for fed-batch bioreactor cultures.

Hypothermic growth can lead to major changes at the physiological level and further effects on metabolic by-products buildup. Lactate production is a key aspect of mammalian cell metabolism [19]. Previous studies showed that lowering culture temperature is accompanied by a reduction of the cell specific lactic acid production rate [20]. Consequently, lactate and glucose metabolism was investigated under different cultivation temperature. The lactate profile shows differences in the level of lactate residual, due to the reduction of culture temperature. Lactate was accumulated in the higher rates in response to changes in temperature from 37°C to 29°C and 28°C than the cultures with a shift to mild hypothermia (32°C, 31°C and 30°C) (day 5) (Fig 2A). Then, the lactate levels declined gradually following cell adaptation and continued during the mid-stationary phase (lactate consumption). Additionally, it was observed that the magnitude of decrease in lactate production was increased at the last days of all cultures, resulting in a final lactate concentration of 13.7 mM and 12.5 mM through the runs with temperature shifting to 29°C and 28°C (respectively), compared to 18.4 mM, 20 mM, 22 mM and 25.8 mM at the mild hypothermia conditions (30°C, 31°C, 32°C and control). Therefore, the final accumulation was lower in hypothermia conditions (Fig 2A), indicating that the lactate reduction may be caused by decreased glucose uptake rate (mostly due to the cell metabolism reduction (Fig 2B)). Meanwhile, the similar pattern of osmolality was observed among all culture conditions.

### Preliminary temperature shift assay (biphasic cultivation): Productivity and misfolding levels

In order to determine the effect of temperature reduction on the protein expression, cell culture supernatant were analyzed for protein titer. It has been previously reported that temperature reduction can favor protein production [21]. Antibody productivity showed a slight improvement with the temperature shifted culture from 37°C to 30°C producing nearly 8% higher cell specific productivity of 22.35 pg/cell/day than value produced under control condition (20.44 pg/cell/day) (Table 2). As a result, an approximately 12% increase in maximum protein concentration was achieved by lowering the culture temperature to 30°C, while a decrease in product expression was observed after shifting the temperature to 29°C and 28°C.

The effects of varying temperature shifts on the production of misfolded Fc-fusion protein is also investigated. Apparently, protein folding enhances in response to low temperature. Temperature downshift to 30°C and 29°C led to about 1.5-fold (35%) reduction in protein misfolding. The lowest level of misfolded protein was achieved at 28°C with at least 50% reduction compared to the control condition (culture temperature of 37°C). It could be concluded that biphasic cultivation may favor protein folding at lower temperature, while productivity enhances under mild hypothermic conditions.

### Preliminary temperature shift assay (biphasic cultivation): Effect of temperature shift timing on productivity and misfolding levels

To explore whether the timing of temperature shift would affect the protein expression and misfolding level, temperatures were shifted to 30°C and 28°C (according to the highest protein expression and lowest protein misfolding level, respectively (section 3.2)) during the log phase of growth (days 3, 5 and 7). It is observed that shifting the temperature within the early-logarithmic phase may negatively affect protein expression. For example, at 30°C by shifting on day 7, protein expression was increased around 19% as compared with the culture shifted on day 3 (Table 3). In contrast, misfolding levels were similar in the temperature shifted cultures during the different days of logarithmic phase. Similarly, the cells that were temperature shifted to 28°C displayed a time-independent pattern of misfolding levels, whereas the expression was slightly increased as time of shifting increased. These results suggest that time of temperature shift during the logarithmic phase would influence protein expression but not protein folding.

**Table 3:** Differential expressions and misfolding levels between temperature shifted cultures during the exponential phase of growth (days 3, 5 and 7).

Time of shifting	Day 3		Day 5		Day 7	
Cell-culture temperature	37°C-30°C	37°C-28°C	37°C-30°C	37°C-28°C	37°C-30°C	37°C-28°C
Protein final titer (mg/L)	1336±24	723±38.5	1500±50	1074±37	1644±28.5	1238±14.5
Misfolding level (%)	33.3±0.42	22.5±0.2	32.7±0.49	22.4±0.42	32.6±0.28	21.8±0.70

### Three-phasic cultivation: Optimization of temperature and time of shifting using RSM

The results of the preliminary study demonstrate that culture temperature can be correlated with protein expression and misfolding levels. It is obvious that misfolding levels were reduced at lower growth temperature. According to these results, reduction of culture temperature to 28°C is effective for correct folding of proteins. However, the productivity was decreased significantly under hypothermic conditions (29°C and 28°C). In contrast, protein expression were gradually enhanced by temperature down shift to 32°C, 31°C and 30°C during the logarithmic phase. Furthermore, the results of biphasic cultivation showed that protein expression depends on time of temperature shift during the logarithmic growth phase although, misfolding levels were time-independent. On the other hand, protein expressed at higher rates during the stationary phase, leading to incorrect folding of proteins [22]. Thus, temperature reduction within the stationary phase can be effective for more accurate folding of proteins.

Therefore, it could be proposed that three-phasic cultivation (two-step temperature downshift) may be efficient in reducing misfolded Fc-fusion protein, while having minimal effects on productivity. The first temperature shift must be applied to balance the beneficial effects of high cell density with high protein productivity. The second temperature shift was chosen to decrease protein misfolding levels. In this strategy, the fed-batch cell culture duration are divided into three distinct stages. The first stage of culture (37°C) enhances cell proliferation, the second stage (32–30°C) promotes protein expression and the third one (28°C) decelerate protein synthesis (due to the high levels of protein expression during the last days of cultivation). Therefore, a slower expression rate is expected to facilitate the accurate folding of proteins. Thus, statistical methods applied to estimate the optimum points for temperature shifting.

RSM was performed to obtain the optimal temperature for first shifting and suitable time. Different combination of first temperature shift level (32°C, 31°C and 30°C), first temperature shift time (72 h, 120 h and 168 h) and time point at which to shift the culture temperature to 28°C (192 h, 240 h and 288 h) as second temperature shift were used for predicting optimal responses (productivity and protein misfolding levels). Temperature shifting to 28°C was selected according to the results of preliminary thermal shift screening with the lowest protein misfolding level. Table 4 presents various combinations used in this design.

**Table 4:** Composition of various experiments of the BBD for independent variables and responses. Factor A: First temperature shifting (°C). Factor B: Time of first temperature shift (h) Factor C: Time of second temperature shift (h). Response 1: Final protein titer (mg L^−1^). Response 2: Protein misfolding level (%).

Run no.	Factor A	Factor B	Factor C	Response 1		Response 2	
				Actual	Predicted	Actual	Predicted
1	31	120	240	1400.0	1411.25	30.0	30.24
2	31	120	240	1350.0	1382.0	30.50	30.24
3	30	168	240	1750.0	1741.25	26.70	26.65
4	30	120	288	1800.0	1792.50	25.50	26.01
5	32	120	192	1310.0	1317.50	31.22	30.86
6	30	120	192	1540.0	1545.00	34.36	34.43
7	32	72	240	1210.0	1218.75	29.80	29.86
8	30	72	240	1400.0	1382.0	31.80	31.27
9	32	120	288	1410.0	1382.0	33.5	33.43
10	31	168	192	1420.0	1423.75	32.0	31.98
11	31	168	288	1670.0	1686.25	27.60	27.28
12	31	72	288	1380.0	1376.25	30.0	30.02
13	31	120	240	1390.0	1382.0	30.70	30.24
14	31	72	192	1320.0	1303.75	31.20	31.66
15	31	120	240	1360.0	1382.0	30.05	30.24
16	31	120	240	1410.0	1405.00	30.05	30.24
17	32	168	240	1330.0	1318.75	31.70	32.23

A wide range of protein titer from 1210 to 1800 mg L^−1^ and protein misfolding level from 25.5 to 34.36% was observed under the investigated growth conditions, indicating the necessity of determining the optimal condition (Table 4).

#### Model fitting and analysis of variance (ANOVA)

The experimental data obtained under different operational conditions were analyzed by the design expert software. The second-order polynomial models representing the final titer (R1) and protein misfolding level (R2) as a function of variables in the experimental region studied are expressed by the following equations:

$$R1=+1382.00-153.75A+107.5B+83.75C-57.5AB-40.00AC+47.5BC+54.00A^{2}-13.5B^{2}+79.00C^{2}$$

$$R2=+30.24+0.98A-0.6B-1.52C+1.75AB+2.79AC-0.8BC+0.35A^{2}-0.59B^{2}+0.55C^{2}$$

Where A, B and C are coded values of temperature for first shifting, time of first temperature shift and time of second temperature shift, respectively.

The significance of the models were analyzed using ANOVA. According to analysis of variance, it can be deduced that the predicted models show quadratic correlation and represent the relationship between the independent variables and response variables.

The model F-values of final protein titer (83.20) and protein misfolding level (45.88) imply that the models are significant at 0.01% level (Table 5). Besides, the lack-of-fit value of 0.6694 and 0.1917 for R1 and R2, respectively would indicate that the error of the models are negligible (*p* >0.05).

**Table 5:** ANOVA results for response surface quadratic models for final protein titer (mg L^−1^) (Response 1) and protein misfolding level (%) (Response 2).

**Response 1**		Sum of Square	Degree of Freedom	Mean Square	F valu*e*	*P* value	
	Model	4.070E+005	9	45226.90	83.20	0.0001	Significant
	Residual	3805.00	7	543.57			
	Lack-of-fit	1125	3	375.00	0.56	0.6694	Not Significant
	Pure error	2680.00	4	670			
	Cor total	4.108E+005	16				
**Response 2**	Model	78.13	9	8.68	45.88	0.0001	Significant
	Residual	1.32	7	0.19			
	Lack-of-fit	0.87	3	0.29	2.57	0.1917	Not Significant
	Pure error	0.45	4	0.11			
	Cor total	79.45	16				

As denoted in Table 6, individual variables had significant linear (A, B and C) and quadratic effects (A^2^ and C^2^). Besides, interactions between variables were also significant for protein production (*p* <0.05). ANOVA analysis of protein misfolding level (%) reveals that the first order, the second order of all individual variables, except A^2^ (first temperature shift level) (*p* = 0.1403), and all interactions had strong effects on measured response. The *p*-value of statistical significance was adjusted to 0.05, which means that all factors with *p*-values higher than 0.05 are non-significant.

**Table 6:** ANOVA of response surface methodology variables for Response 1 (final protein titer (mg L^−1^)) and Response 2 (protein misfolding level (%)).

		Response 1			Response 2	
	Mean Square	F-valu*e*	*P-*value	Mean Square	F-valu*e*	*P-*value
A	1.891E+005	347.91	<0.0001	7.72	40.81	0.0004
B	92450.00	170.08	<0.0001	2.88	15.22	0.0059
C	56112.5	103.23	<0.0001	18.54	98.01	<0.0001
A^2^	12277.89	22.59	0.0021	0.52	2.77	0.1403
B^2^	767.37	1.41	0.2735	1.48	7.81	0.0267
C^2^	26277.89	48.34	0.0002	1.29	6.79	0.0351
AB	13255.00	24.33	0.0017	12.25	64.74	<0.0001
AC	6400.00	11.77	0.011	31.02	163.97	<0.0001
BC	9025.00	16.6	0.0047	2.56	13.53	0.0079

The high coefficient of determination of both models with the R^2^ values of 0.9907 and 0.9833 for response 1 (final protein titer) and response 2 (protein misfolding level) respectively, indicate that the experimental data are fitted into the predicted models. The analysis of adjusted and predicted coefficients of determination which should be greater than 0.6 [23] implies that the majority of responses can be described with the predictive statistical models. Furthermore, the adjusted *R*^2^ value of 0.9788 is in reasonable agreement with the predicted *R*^2^ value of 0.9460 for protein titer. Similarly, adjusted *R*^2^ value of 0.9619 is in reasonable agreement with predicted *R*^2^ value of 0.8154 for protein misfolding level.

The value of the signal to noise ratio, adequate precision, greater than 4 is desirable, [24] which in this case the adequate ratios were obtained (32.086 for response 1 and 25.823 for response 2), indicating that the background noise levels are lower than the measured values for responses [23].

**Fig 3:**
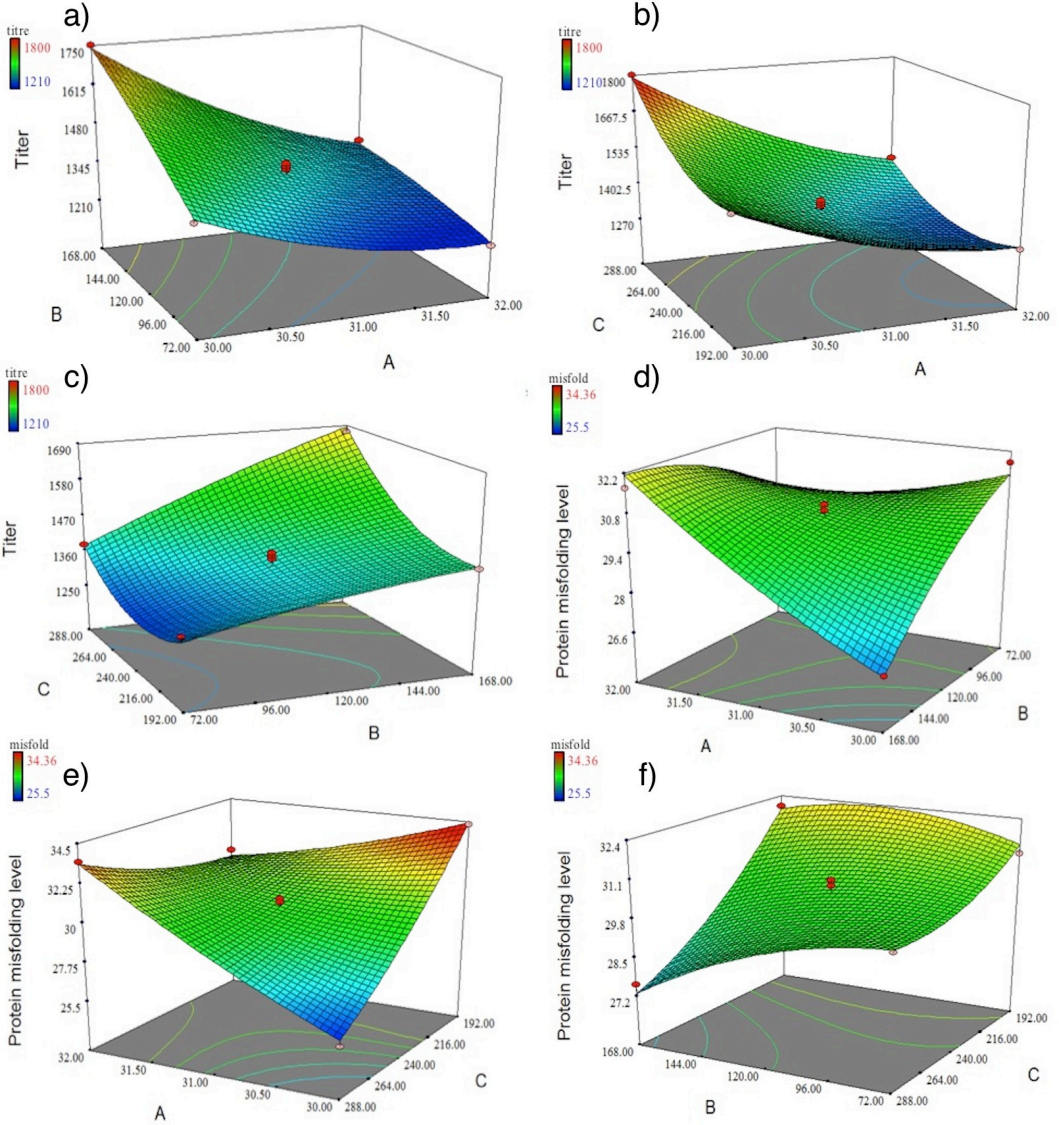
Response surface for Fc-fusion protein titer and protein misfolding level as a function of temperature (°C) and first temperature shift time (h) (a and d), temperature (°C) and second temperature shift time (h) (b and e) and first temperature shift time (h) and second temperature shift time (h) (c and f). A: temperature (°C), B: first temperature shift time (h) and C: second temperature shift time (h).

The three-dimensional response surface plots and two-dimensional contour plots were used to illustrate the effects of the chosen parameters to obtain optimal amounts of correctly folded Fc-fusion protein as shown in Fig 3. In each sketch, two factors changed within the range of experimental data, while the other one kept constant at the center point.

The overall analysis thus demonstrated that the proposed models describing the product titer value and protein misfolding levels on the 15th days of cultivation are significant and can be applied for predicting and optimization purposes within the characterized design space.

#### Effect of parameters on productivity and misfolding level

Based on the Fig 3A and 3B, lower levels of temperature favors higher expression of the protein. Contour plot shows the titer predicted probability of about 1400 mg L^−1^ by changing the temperature from 37°C to 30°C on day three (72 h), while ~1200 mg L^−1^ titer value by changing the temperature from 37°C to 32°C at the same time. Moreover, the highest level of protein production was attained when culture temperature shifted in the mid-late logarithmic growth phase (168 h) instead of early logarithmic growth phase (72 h). Contour plot of the predicted values estimates the titer of ~1700 mg L^−1^ when temperature shifted to 30°C at 168 h and titer value of ~1400 mg L^−1^ following temperature shift to 30°C at 72 h. As shown, the highest amount of protein expression was occurred at shifting the temperature to 30°C at 168 h.

Fig 3C shows correlation of first and second temperature shift time with protein production. It can be observed that the increase in protein expression occurred with both increase in first and second temperature shift time. Furthermore, the elliptical contour plot indicated the significant interactions between the corresponding variables [25].

The combined effect of first temperature shift–first temperature shift time, first temperature shift -second temperature shift time and first temperature shift time-second temperature shift time on protein misfolding level is shown in (Fig 3D, 3E and 3F), respectively.

In Fig 3D the dependency of the protein misfolding levels and first temperature shift has been shown. According to this figure, shift of the culture temperature from 37°C to 30°C resulted in enhancement of protein folding. The lowest level of misfolding level was attained after temperature shifting to 30°C at 168 h. Furthermore, there is an interaction between the times of two thermal shift. Increasing both first and second temperature shift times have a favorable effect on protein folding. As shown in Fig 3E exposure to cold stress by temperature downshift to 28°C during the mid-stationary phase resulted in lower misfolded protein level. According to the plot 3f, misfolding level was decreased to around 26% by steadily increased of second temperature shift time.

Further, the point prediction feature of RSM determined the optimal value of first temperature shift to 30°C at 155 h and second temperature shift to 28°C at 281 h for maximum productivity (1878 g/L) and minimum misfolding level (24%).

**Fig 4:**
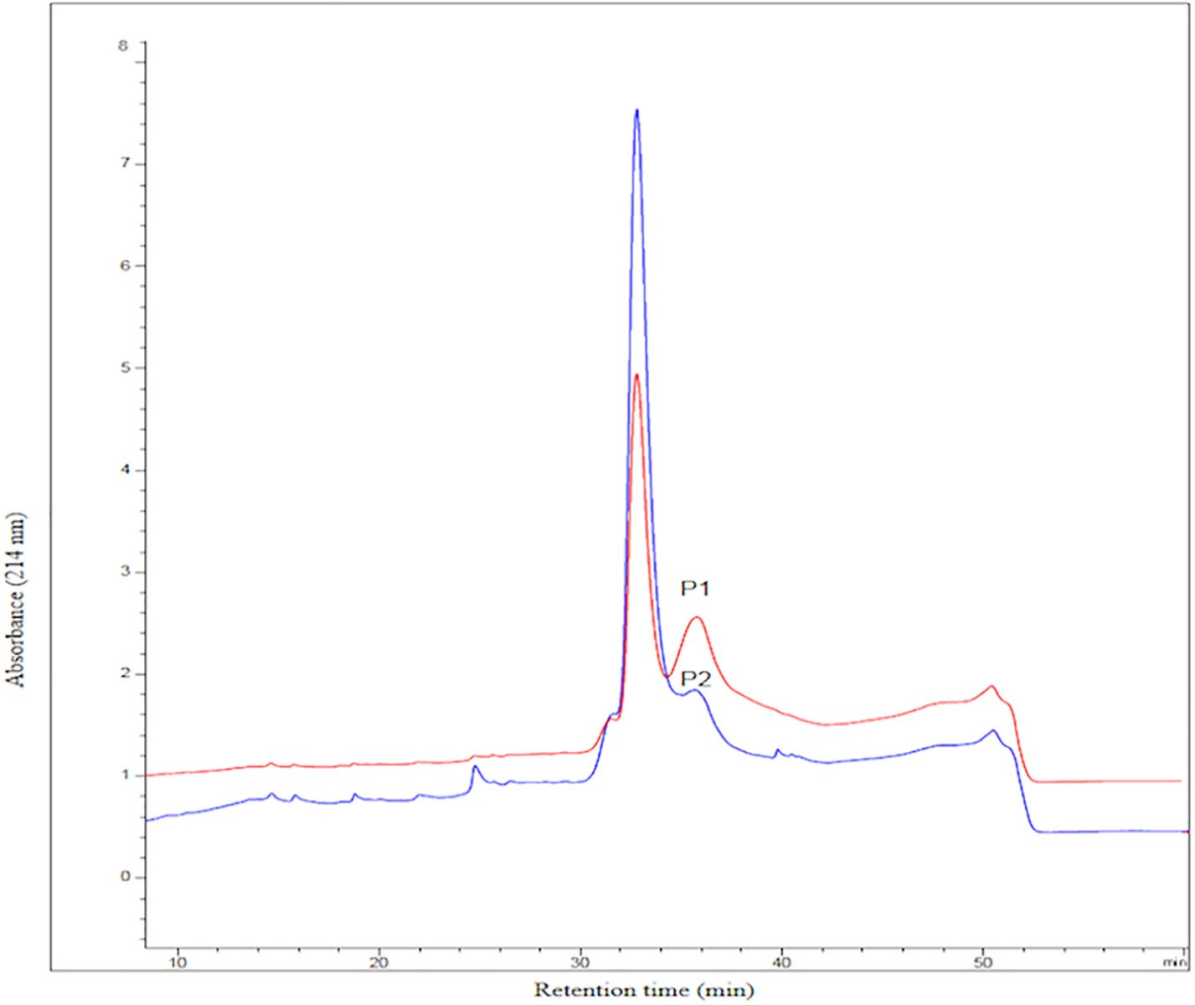
Elution profile of Fc-fusion protein on a butyl NPR HIC-HPLC column. Optimized condition (three-phasic cultivation) results in a decrease of post-main-peak (P2) compared to the control condition (P1). Control condition was carried out at physiological temperature (37°C).

HPLC profile of the FC-fusion protein eluted from HIC-HPLC column is represented in Fig 4. The misfolded proteins eluted after the main peak. It is obvious from this figure that applying two- temperature shift to gradually decrease the temperature was resulted in smaller post-main peak (represented as P2 peak) compared to the control condition performed at 37°C.

### Validation and confirmation of the RSM model

The optimal condition for maximum Fc-fusion protein titer (1878 mg L^−1^) with minimum level of protein misfolding (24%) was predicted in 5-L bioreactor by shifting the temperature to 30 and 28°C respectively after around 6 and 12 days of cultivation (155 and 281 h). The validation experiments were conducted by running three consecutive 5 L scale batches under predicted condition. The appropriateness of the process optimization was deduced from close correlation between predicted values of the model and experimental data which were statistically analyzed (*p* <0.05).

To further determine the efficiency of the model, large-scale experiments (30 and 250-L bioreactors) were conducted. The results showed a good agreement with previous data and confirmed the validity of the model (*p* <0.05) (Table 7).

**Table 7:** The large-scale experiments under optimal condition.

Bioreactor	Protein titer	Misfolding level
30-L	1883 ± 70.94	25.4 ± 1.08
250-L	1844 ± 45.09	25± 2.00
Predicted value	1877.98	24.00

## Discussion

The Chinese hamster ovary cell (CHO) has been widely used as a primary platform in the biopharmaceutical industry for the past two decades. Low yield and other limitations, however, have stimulated the need to improve or optimize the bioprocess design to meet the global market demand [26]. On the other hand, the vast majority of pharmaceutical products that require a high degree of purity are isolated by a series of downstream processing techniques which are affecting protein final yield [27]. Expression of intact and properly folded proteins favorably improves productivity by reducing the number of purification steps [28]. Therefore, the aim of this study was to determine the optimal condition for enhancement the correctly folded Fc-fusion protein production while improving protein yield. Fc-fusion protein, a recombinant dimer protein with a molecular weight of 150 KDa, is a genetically engineered humanized protein and effective in treatment of various autoimmune disease.

Biphasic cultivation has been previously proposed as an effective strategy for maintaining cell proliferation while increasing the protein expression capability [29]. Furthermore, the expression under hypothermic conditions has been reported to have a positive effect on reducing misfolded proteins at the cost of lower protein expression [30]. Despite the large number of studies indicating positive impacts of single temperature shift on protein productivity or misfolding level, no experimental approach has addressed the effect of three-phasic cultivation on improving protein productivity and folding simultaneously [31–33]. In this study, preliminary biphasic temperature shift experiment was performed as a pre-optimization study to monitor the effect of culture temperature on growth kinetics, protein titer and misfolding level of the fusion protein.

Kinetic assay revealed that the temperature downshift can result in lower Max VCC. Previous studies suggested that cells fail to undergo the G(2)/M transition at or below a critical temperature [34]. Furthermore, the specific growth rate (μ) seems to be affected for cultures exposed to hypothermia (Table 2). Reduction of metabolism could be associated with decrease in specific growth rate. Moore et al. reported that overall rate of metabolism was reduced as a result of temperature reduction to 30°C. [35]. However, cell viability usually increases following temperature downshift by reducing cell metabolism, delaying cell death and extending RNA half-life. Such effects have been related to prolong stationary phase culture and therefore product yield improvement [36]. Furthermore, it has been demonstrated that mild hypothermia condition improves productivity through the general responses involving the cell cycle, transcription and translational regulatory, and the cytoskeleton arrangement [37]. This is in agreement with the data obtained in this study which has shown that temperature downshift to 30°C gave the highest protein final titer. Moreover, IVCC and specific production rate (Qp) during culture are considered to be a critical output for high volumetric product yield in the commercial biomanufacturing [38]. IVCC is usually mirrored by the synergistic effects of cell specific growth rate and culture longevity [39]. Yoon reported that the final IVCC at 33°C was lower than control condition (37°C), despite the increased culture period [11]. However, in this study culture duration had effect on IVCC and the highest one was achieved during a temperature shift to 32°C.

Lactate accumulation has been observed to adversely affect the cell density, product yield and quality [26]. Lowering culture temperature is one of the four common methods for reducing lactic acid production [20]. It has shown that approximately 74% of the lactate produced during the exponential phase of growth was consumed in late stage of cultivation at 32°C compared to 12% at physiological temperature [40]. In this study, lactate accumulation induced by temperature shifting to 29°C and 28°C on the day of temperature shift. Cold shock leads to an immediate increase of glucose uptake than required for normal cell metabolism resulted in lactate accumulation [41]. Although, the lactate levels declined following cell adaptation, confirming the beneficial effects of hypothermic condition on lactate consumption.

A common limitation encountered in large-scale production of biopharmaceutical especially fusion protein, is that a significant percentage of proteins are produced in misfolded forms which make them prone to aggregation. Therefore, there is a need to find efficient ways to reduce protein misfolding [42]. It is reported that lower temperature results in improved folding and assembly of protein [43]. Additionally, low culture temperature has a significant impact for reducing the degree of molecular aggregation [30]. Hypothermic culturing promotes folding capacity by influencing ER chaperones [44]. Reducing the culture temperature also appeared to enhance the secondary and tertiary structure of expressed proteins [45]. It was reported that the conformation of several misfolded proteins accumulated in ER is improved by low-temperature culturing (26°C) through the regulation of ER chaperones expression [46]. Compos reported that virus-associated E protein expressed in HEK-293T was misfolded at 37°C but correctly folded at 28°C [47]. Here, upon temperature downshift to 28°C, Fc-fusion protein misfolding level has been shown to decrease around two fold in comparison to control condition (37°C). Similar results have been reported for significantly decreased proportion of misfolded TNFR-Fc (Tumor necrosis factor receptor- Fc fusion protein) while cells cultured at reduced temperature of 29.5°C [12].

Time of temperature shift is another critical factor for protein expression and proper folding. Reducing the culture temperature is generally applied during the mid or mid-late exponential phase [48]. Kumar checked the maximal productivity of recombinant proteins through reduction of the culture temperature from 37°C to 31°C after 72 h and 144h. Their results demonstrate the protein type-specific regulation following temperature shift at either 72 h or 144h [49]. The results of the present study showed that reducing the culture temperature during the late exponential phase leads to higher protein expression. Reaching the maximum cell density plays a role for this observation. Meanwhile, reducing the culture temperature during the exponential phase has not advantage of the correct folding of protein.

According to the results of single temperature shift cultures, although the level of misfolded product were mostly decreased by single temperature downshift to 28°C, the final titer was 30% lower than the maximum value obtained for temperature shifting to 30°C. Therefore, the combination of two temperature shifts has been assumed to be effective for decreasing the amount of protein misfolding, without affecting productivity. In order to study the interaction of first temperature shift during the exponential phase (early, mid and late) and second temperature shift to 28°C at early to late stationary phase, RSM was performed. RSM can serve as a successive approach for modeling and optimization of multiple parameters even with complex interactions [50].

It was evident from experimental results that at lower level of temperature, there was a linear increase in protein titer. Meanwhile, the maximum protein titer was recorded by shifting the temperature at the end of the exponential phase. It could be due to delayed induction of growth arrest phase. Similar results were reported where fed-batch cultivation with a temperature shift at the mid-exponential phase gave the best protein production, due to the optimal cell density [51, 52]. Furthermore, the second temperature downshift to 28°C at the mid-late stationary phase was found as the optimum point for misfolding level while having minimal effects on protein titer. It is in agreement with Gomes's report which showed that culture temperature above 30°C can lead to increased protein misfolding/ aggregation and thus production of nonfunctional product [12].

Generally, protein production in mammalian cell line is greater in stationary phase of growth when there is no competition with endogenous cellular processes [9]. In this study, Fc-fusion protein production reached its maximum level in the last days of culture, it may increase the chance of improper folding during the final stage of culture. It demonstrates the effect of reduced culture temperature to obtain optimal folding through the mid-stationary phase.

According to these results, three-phasic cultivation for the highest accumulation of correctly folded protein is estimated. It can be explained that gradually reducing the temperature is related not only to the maximum productivity but also to the correct folding. The first step of temperature shifting during the mid-late exponential phase resulted in high cell densities and productivity. The second temperature shift to 28°C induces properly folded production of proteins.

## Conclusion

In this study, the combination of three-phasic cultivation method by applying two temperature downshifts in fed-batch experiments were established using design of experiments (DOE) methods. According to the results, combination of two temperature shifts for the highest and correctly folded Fc-fusion protein is estimated. Regarding the significant portion of misfolded protein production in large-scale therapeutic protein manufacturing, the three-phase cultivation can be applied for decreasing the amount of protein misfolding, without affecting productivity. The optimized setting resulted in 25% increased productivity and 50% decreased misfolding level compared to the control condition (37°C) and about 14% increased productivity and 27% decreased misfolding level compared to the one step temperature downshift to 30°C. Furthermore, pilot scale studies confirmed the effectiveness of the method. To our knowledge, this is the first report of three-phasic cultivation to simultaneously improve productivity and protein folding. This method suggests a cost effective approach to enhance product yield while maintaining quality.

## Supporting information

S1 Text The methodology for optimized expression of misfolding-prone proteins. (PDF)

## Abbreviations

ANOVA: analysis of variance
BBD: Box-Behnken design
CHO: Chinese hamster ovary cell
DO: dissolved oxygen
DOE: design of experiments
ELISA: enzyme linked immunosorbent assay
HIC-HPLC: hydrophobic interaction chromatography-high pressure liquid chromatography
IVCC: integral of viable cell concentration
Max VCC: maximum viable cell concentration
PBS: phosphate-buffered saline
PTMs: post-translational modifications
RSM: response surface methodology
RWCB: research working cell bank
