# Supplementary figures and images for: Therapeutic Fc fusion protein misfolding: A three-phasic cultivation experimental design
Source: PLoS One. 2019 Jan 16;14(1):e0210712. doi: 10.1371/journal.pone.0210712 (PMC6334962; doi:10.1371/journal.pone.0210712)

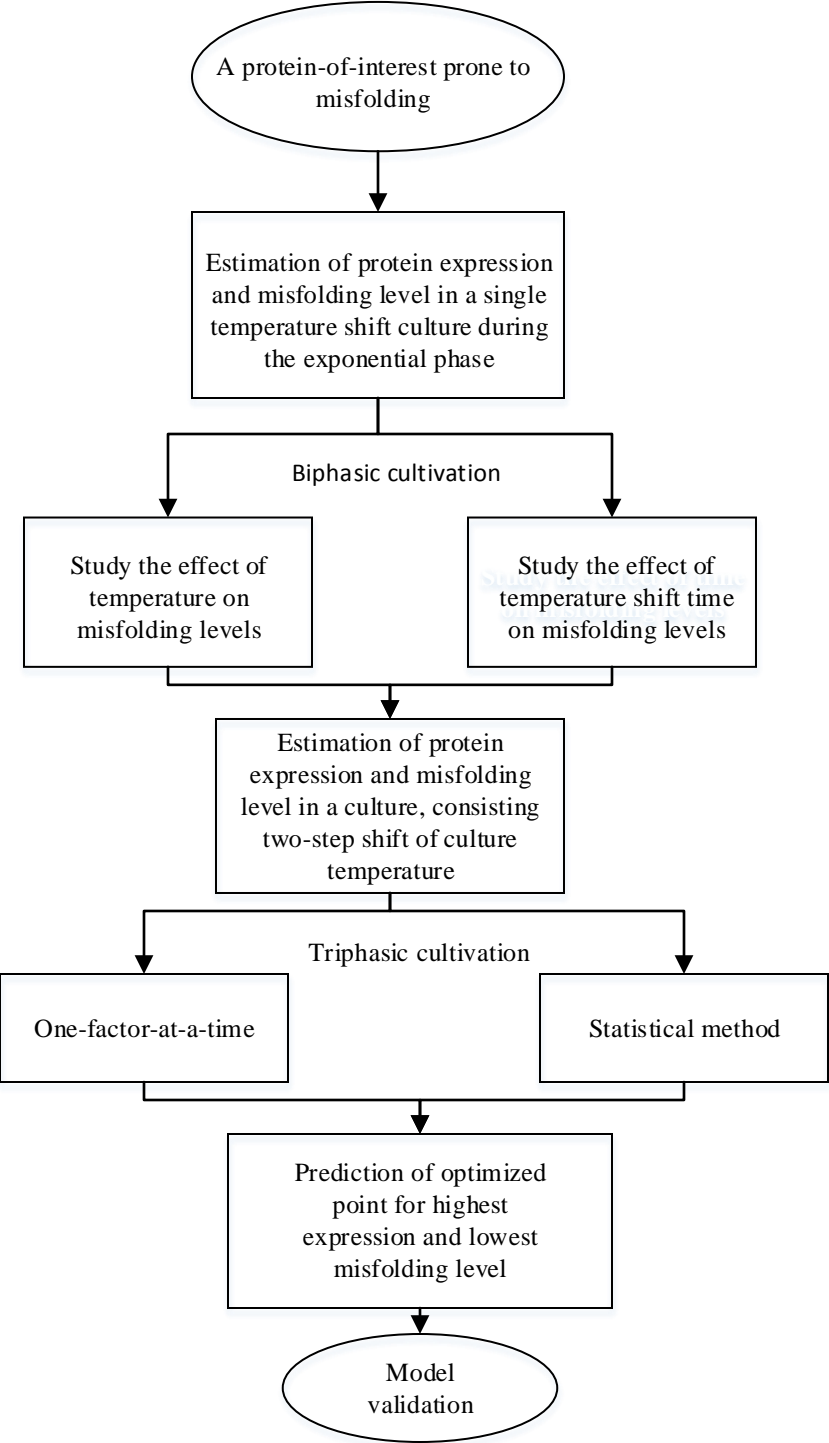

Supplement: S1 Text — (PDF) [file pone.0210712.s001.pdf]
